# Admissions Profiles, Academic Stress, and Student Outcomes in Veterinary Education: A Narrative Review

**DOI:** 10.3390/vetsci13030235

**Published:** 2026-02-28

**Authors:** Ihab Habib

**Affiliations:** Department of Veterinary Medicine, College of Agriculture and Veterinary Medicine, United Arab Emirates University, Al Ain 15551, United Arab Emirates; i.habib@uaeu.ac.ae

**Keywords:** veterinary education, student well-being, academic stressors, coping strategies, professional development

## Abstract

Veterinary education is academically demanding and emotionally intensive, affecting student performance, well-being, and long-term professional development. This narrative review synthesizes current evidence on academic stressors, admissions predictors, coping mechanisms, and institutional responses in veterinary training. Cognitive indicators such as Grade Point Average (GPA) and standardized test scores reliably predict early performance in pre-clinical biomedical courses. However, these measures do not adequately capture essential non-cognitive attributes, including resilience, adaptability, motivation, and communication skills, which are critical for sustained success in clinical environments. Holistic admission approaches show promise but remain inconsistently validated across institutions. Academic stress in veterinary programs arises from heavy curricular loads, frequent high-stakes assessments, financial pressures, and transitions into clinical training. Persistent stress exposure is associated with anxiety, depressive symptoms, and burnout risk. Evidence suggests that structured wellness initiatives, peer mentoring, and resilience-building programs can mitigate these effects when embedded systematically within the curriculum. Current literature is largely cross-sectional and geographically concentrated in Western educational contexts, limiting causal inference and generalizability. Longitudinal, multi-institutional research linking admissions profiles to academic trajectories and psychological outcomes is needed. Integrating cognitive and non-cognitive evaluation with sustained institutional support may enhance retention, academic performance, and professional preparedness in veterinary education.

## Simple Summary

Veterinary education is academically demanding and emotionally challenging, yet it remains unclear how student selection, academic stress, and well-being interact over time. This review explores how admission characteristics, training pressures, and institutional support influence student outcomes. Strong prior academic performance predicts early success in science-based courses, but grades alone do not capture important qualities such as resilience, adaptability, motivation, and communication skills, which are essential in clinical training. Students commonly face heavy workloads, frequent examinations, financial strain, and stressful clinical transitions. Ongoing stress is associated with anxiety, low mood, and burnout risk. Evidence shows that programs embedding mentoring, wellness training, peer support, and accessible counseling within the curriculum can improve well-being and engagement. A balanced approach that considers both academic readiness and personal strengths, combined with sustained institutional support, may enhance student retention, academic achievement, and long-term professional sustainability.

## 1. Introduction

Veterinary education constitutes a highly demanding environment where students are continually exposed to complex academic and emotional pressures [1]. Stress within this context is multifaceted, encompassing heavy curricular loads, frequent examinations, and competitive grading systems, alongside transitional challenges associated with adapting to the professional school setting [2,3]. Although such pressures are shared across many health professions, their intensity may be amplified in veterinary programs due to the expansive knowledge required across multiple species and disciplines, as well as prolonged practical training. Recent surveys confirm that veterinary students experience elevated stress and depression rates compared to peers. Nearly two-thirds report at least mild depression, with stress levels correlating strongly with depressive symptoms [4]. These findings align with reports from other countries that veterinary students face worse mental health outcomes than the general population [5]. Together, this evidence underscores the urgency of addressing student well-being from day one of training.

These academic demands within the veterinary curriculum are further intensified by the emotional responsibilities of patient care and client communication, which require sustained empathy despite evidence of gradual empathic decline during training [6]. The cumulative effect of academic and emotional pressures is reflected in elevated rates of depression and anxiety, reduced life satisfaction, and persistent burnout risk across all stages of veterinary study [3,6].

These observations raise important questions regarding how admissions characteristics relate to subsequent resilience and preparedness. Stronger pre-admission academic foundations may buffer the impact of stress on early performance [7,8], yet academic readiness alone does not fully explain variation in well-being trajectories. This underscores the need to evaluate whether admissions frameworks should extend beyond immediate academic competitiveness to include adaptability and coping potential. Given the multidimensional nature of veterinary training—from entry through clinical transitions and into professional practice—single-domain predictors are unlikely to capture long-term outcomes [8]. Accordingly, contemporary scholarship calls for longitudinal research linking selection criteria with later academic performance and psychological well-being [9]. Such evidence would support refinement of admissions and curricular policies in ways that protect student welfare while maintaining professional and academic standards.

Despite increasing recognition of mental health challenges in veterinary training, important conceptual gaps remain. Admissions processes continue to rely heavily on cognitive indicators such as GPA and standardized test scores, while evidence suggests that non-cognitive factors may influence resilience, stress adaptation, and long-term outcomes [10]. At the same time, much of the existing research is cross-sectional and institution-specific, limiting understanding of how admissions characteristics, stress exposure, and institutional support interact over time. A clearer synthesis of these intersecting domains is needed to inform evidence-based admissions reform and curriculum design. The aim of this narrative review is to synthesize current evidence on academic and non-academic stressors affecting veterinary students, examine cognitive and non-cognitive predictors of academic success, and evaluate how admissions criteria, coping mechanisms, and institutional factors interact to influence student well-being and professional readiness within veterinary education.

## 2. Literature Identification and Scope of Review

This review adopts a narrative synthesis approach intended to integrate conceptual and empirical developments across veterinary education research rather than to provide a systematic meta-analysis. Relevant literature published between 2005 and 2025 was identified through searches of PubMed, Scopus, and Web of Science using combinations of keywords including “veterinary students,” “academic stress,” “admissions predictors,” “non-cognitive traits,” “resilience,” “burnout,” and “well-being.” Reference lists of key articles were also screened to identify additional relevant studies.

Inclusion criteria comprised peer-reviewed empirical studies and review articles focusing on veterinary education contexts and reporting associations between admissions characteristics, stress exposure, academic outcomes, or psychological well-being. Studies conducted in other health professions were considered selectively where conceptually informative.

As a narrative review, formal quality appraisal, protocol preregistration, and systematic exclusion procedures were not applied. While this approach allows thematic integration and conceptual exploration, it may limit reproducibility and completeness of the evidence base. Findings should therefore be interpreted as an informed synthesis rather than an exhaustive systematic assessment.

## 3. Key Academic and Non-Academic Stressors

Literature consistently identifies academic demands, including heavy coursework and frequent examinations, as major sources of stress among veterinary students. First-year cohorts, in particular, report anxiety related to overwhelming study loads, with those lacking prior science preparation facing greater difficulty [11]. Non-academic pressures such as financial strain and social isolation further exacerbate distress; in Austria, over half of surveyed students exceeded moderate depression or anxiety thresholds, attributed partly to debt and poor work–life balance [5]. Gender-based differences are also evident, with female students more frequently reporting heightened stress and anxiety, likely reflecting intersecting cultural and workload factors [4]. Longitudinal studies reveal that such pressures often persist or intensify as students advance into clinical training [3,12], underscoring that veterinary student stress is a cumulative and enduring phenomenon across the curriculum.

Table 1 integrates findings from multiple studies by comparing academic and non-academic stressors, emphasizing their prevalence and potential downstream consequences. A recurring limitation in the literature is the absence of longitudinal research that follows how early risk factors, such as limited social support at entry or low baseline financial security, interact with increasing curricular demands over time. Another insufficiently examined aspect is the influence of demographic variables on stress exposure patterns. Gender differences, for instance, are consistently reported in the prevalence of anxiety symptoms among veterinary students [6], yet the underlying mechanisms remain debated. It is still uncertain whether these disparities arise from biological predispositions to affective disorders or from sociocultural influences that shape help-seeking behaviors differently across genders.

Institutional strategies to address student stress display substantial heterogeneity, ranging from the integration of structured wellness curricula within the core timetable to reliance on optional counseling services made available only upon student request [28]. Such variability complicates efforts to benchmark what constitutes an effective mitigation approach. Evidence indicates that when proactive support, such as peer mentoring programs or staged transitions into demanding clinical rotations, are implemented, students report lower levels of perceived overwhelm compared to cohorts without comparable scaffolding [27]. Literature increasingly positions veterinary student stress not as an inevitable consequence of professional rigor but as the emergent outcome of an interacting system that encompasses curricular design, individual coping resources, institutional support mechanisms, and broader socio-economic contexts [3,15]. Systematic refinement across multiple leverage points, including admissions weighting, curricular pacing, and structured psychosocial support, offers a viable pathway to reducing cumulative stress exposure while maintaining the integrity of educational standards.

## 4. Coping Mechanisms and Resilience Building

Efforts to address the mental health challenges inherent in veterinary training are increasingly focused on fostering adaptive coping strategies that can buffer students against the diverse stressors outlined in the previous section. Research consistently highlights the value of responses such as systematic problem solving, proactive help-seeking from peers, mentors, or faculty, and the maintenance of healthy routines encompassing adequate sleep, regular exercise, and balanced nutrition [29,30]. In addition, the deliberate preservation of non-academic interests and social relationships has been shown to provide psychological distance from academic pressures and to reinforce a sense of personal identity beyond professional training [26]. Collectively, these adaptive behaviors are associated with reductions in perceived stress load, greater resilience to academic and clinical demands, and improved self-reported well-being indices [15]. Importantly, cultivating such strategies is not merely an individual responsibility but is increasingly viewed as a shared institutional mandate.

Universities that integrate wellness training, structured peer-support systems, and accessible counseling into the formal curriculum appear better positioned to normalize adaptive coping, reduce stigma around help-seeking, and embed resilience-building as an educational objective on par with technical competence. Evidence points to the importance of formally embedding awareness and skill-building for coping into the curriculum itself. Structured interventions, such as targeted sessions on identifying personal stress triggers or guided planning of stress management plans, introduced at orientation and revisited periodically yield measurable benefits in veterinary cohorts [15,24]. Orientation phases have been leveraged by some institutions to combine community-building activities with facilitated discussions on coping resources, including mentorship introductions and exposure to campus mental health services [31]. Intentional creation of peer-support opportunities, e.g., lunch forums or facilitated discussion groups, offers structured environments to share challenges without fear of stigma [15]. Such environments also allow for communal reframing of difficult events (e.g., unsuccessful patient outcomes) which might otherwise leave individual students isolated in their appraisal.

Table 1 summarizes selected coping mechanisms reported by veterinary students alongside evidence on observed impacts on mental health and academic functioning. Diversity across studies reflects differences in measurement tools and intervention intensity but provides a broad comparative view amenable to curriculum design considerations. Adopting a healthy lifestyle appears to mitigate psychological distress among veterinary students. Regular physical activity, adequate sleep, and time spent outdoors are consistently associated with lower levels of stress, anxiety, and depression, with even brief daily exercise or outdoor exposure (such as 30 min per day) linked to significantly reduced symptom scores [19].

Institutional culture shapes whether such coping tools are treated as optional extras or acknowledged necessities. Where leadership explicitly endorses wellness initiatives, through allocation of faculty time for mentoring or scheduling protected hours for non-academic enrichment, students may perceive greater permission to invest effort into self-care activities without guilt over perceived lost productivity [6]. Overall, while much informal practice around student coping remains reliant on motivated individuals finding what works best for them under constrained circumstances, there is steadily accumulating justification, and ethical imperative, for institutions to constitute resilience building as an equally examinable outcome alongside scientific competencies within contemporary veterinary curricula.

## 5. Predictors of First-Year Success and Risk Factors

### 5.1. Cognitive and Academic Background

Prior academic preparation remains one of the strongest predictors of early performance in veterinary education. Undergraduate GPA (UGPA) and Graduate Record Examination (GRE) scores consistently forecast success in the first year, with UGPA and GRE verbal reasoning together explaining over half of the variance in Veterinary Educational Assessment outcomes, a benchmark of biomedical science mastery [14,32]. Science GPA shows a clear link to biomedical coursework, though its predictive value diminishes in clinically focused years [21,33]. GRE quantitative reasoning adds little beyond GPA, while secondary school GPA is more relevant in non-U.S. contexts at earlier academic stages [34]. Rigorous prerequisite coursework supports integration of new material but may disadvantage students from less-resourced institutions [35].

These metrics are most powerful during pre-clinical phases, where intensive scientific content dominates learning demands. Yet exclusive reliance on them risks overlooking resilience, adaptability, and communication—competencies critical for clinical success. Students with recent science backgrounds often adapt quickly to laboratory and theoretical work [23], while those entering after study gaps may struggle initially but draw on maturity and broader life experience to build resilience [11]. Prior animal-handling or veterinary assistant experience can ease procedural skill acquisition [36], though it may also reinforce narrow career orientations. Collectively, these findings underscore both the enduring relevance and the limitations of cognitive indicators in predicting long-term success.

### 5.2. Non-Cognitive Predictors

Evidence suggests that first-year veterinary student outcomes are influenced not just by cognitive readiness, but also by dispositional traits such as self-efficacy and internal locus of control [10], as well as psychosocial stressors including academic and transitional stress, anxiety, and depression [3]. Frameworks derived from Sedlacek’s non-cognitive model (developed by William E. Sedlacek (University of Maryland) to evaluate student potential beyond traditional cognitive measures) emphasize variables such as self-efficacy, realistic self-appraisal, motivation, adaptability, and social support [37]. For example, students with strong self-efficacy tend to report lower test anxiety and greater willingness to engage with challenging material, while realistic self-appraisal reduces maladaptive perfectionism and supports quicker recovery from setbacks [37].

Intrinsic motivation, particularly when tied to professional identity, is associated with persistence under stress and reduced burnout risk [38]. Similarly, adaptability and tolerance of ambiguity predict smoother transitions into problem-based learning and clinical contexts where uncertainty is high [34]. Social support utilization also moderates stress effects by facilitating cultural acclimatization and collaborative resource-sharing. However, non-cognitive traits rarely substitute for insufficient cognitive preparation; rather, they appear to buffer against stress and sustain engagement when academic demands peak.

Admissions processes incorporating behavioral interviews, situational judgment tests, or structured scoring rubrics attempt to operationalize these constructs [39]. Yet their predictive validity remains inconsistent due to interviewer bias, self-reporting limitations, and limited longitudinal validation beyond the pre-clinical years. Early curricular interventions may therefore be as important as selection filters, with evidence suggesting that adaptability and communication training can elevate lower-scoring entrants toward parity [15].

Taken together, cognitive and non-cognitive predictors exert complementary influences on early veterinary student outcomes. While UGPA and GRE-type metrics remain the strongest predictors of first-year biomedical science achievement, non-cognitive variables shape resilience, stress management, and sustained engagement. Current evidence highlights a need for longitudinal, multi-institutional datasets linking combined entry profiles to long-term outcomes such as attrition, veterinary licensing examination success, and career satisfaction. Such data would enable refinement of admissions weighting schemes that balance cognitive readiness with psychosocial adaptability, ensuring both academic performance and professional sustainability.

## 6. Interplay Between Stress, Admissions, and Academic Outcomes

The relationship between admissions profiles, stress exposure, and academic outcomes in veterinary education is dynamic and reciprocal rather than linear. Cognitive indicators such as GPA and GRE scores provide reliable baselines for early biomedical performance [14], yet their predictive strength diminishes when students confront complex clinical contexts requiring adaptability and communication [9]. Conversely, entrants selected for interpersonal skills often adapt more effectively to variable demands but may initially require greater support in science-intensive modules before reaching comparable achievement levels [32].

Stress responses mediate the extent to which admission’s advantages translate into sustained success. Financial pressures, relocation, and workload intensification can erode the benefits of strong cognitive preparation, while prolonged assessment schedules may blunt the advantages of non-cognitive strengths such as social resourcefulness [15]. Chronic stress impairs working memory and deep learning, producing feedback loops that degrade both academic performance and psychological resilience [31].

Empirical findings suggest recurring patterns. High pre-admission GPA predicts strong early biomedical outcomes but is less relevant for later tasks reliant on uncertainty management [40]. Non-cognitive factors such as self-efficacy show moderate associations with persistence and reduced burnout, though operationalizing their measurement remains challenging [34]. Motivation quality, particularly intrinsic motivation, supports sustained engagement and mediates persistence through common motivational downturns during training [32]. Table 2 synthesizes literature evidence, contrasting how predictor categories and stressor types converge to influence veterinary student outcomes across the training timeline.

Qualitative accounts add nuance to these aggregate trends by revealing that student perception of support availability can modulate the functional impact of both selection advantages and vulnerabilities. For example, high-achieving entrants accustomed to structured environments may experience disproportionate anxiety when expectations are unclear or feedback delayed; meanwhile, those entering with average scores, but strong peer-networking abilities can leverage group study to mitigate individual content gaps [12]. This illustrates how psychosocial resources act as force multipliers or dampeners for the trajectory set by admissions baselines. Institutional responses shape these interactions substantially.

Programs embedding wellness curricula alongside technical training help maintain adaptive coping practices established pre-entry or introduced early on [31], reducing the likelihood that acute stress moments cascade into chronic impairment. Where cultures implicitly valorize endurance over self-care, even resilient entrants may suppress help-seeking behaviors until difficulties compound beyond easy remediation [6]. The reverse holds true for nurturing educational climates: entrants with modest preparedness can achieve parity through continuous mentorship and accessible remedial pathways without stigma.

Financial context frequently emerges as an intersectional axis within this interplay. Debt load and financial insecurity consume cognitive bandwidth otherwise available for learning tasks [41], acting as chronic background stressors that magnify acute curricular pressures. Students already at higher risk due to lower baseline cognitive preparation or weaker coping repertoires are especially vulnerable here; economic anxiety can trigger disengagement cycles observable in declining grades well before critical failure thresholds are reached [8,41]. An underexplored dimension in existing research is time-dependent variation in predictor–outcome relationships. Many quantitative studies focus narrowly on first-year performance associations without tracking whether early predictors retain salience across didactic–clinical transitions [11,39]. Qualitative data suggests partial shifts: non-cognitive traits like adaptability may grow more determinative once students face unpredictable caseloads and increased client interaction stakes in later years, a stage when purely cognitive predictors lose some explanatory weight for variance in outcomes [10].

From a methodological perspective, isolating causality within this intertwining system is difficult due to confounding loops: stress both results from and contributes to declining academics; conversely, strong academics reduce some sources of stress while generating others (e.g., heightened self-expectations) [12,31]. Longitudinal mixed-method designs are therefore best suited to disentangling sequences within these cycles. Considering these findings, refined admission frameworks could employ hybrid weighting schemes combining validated cognitive metrics with reliably scored non-cognitive assessments tailored for developmental malleability rather than static “fit” judgment [14,34]. Prospective monitoring would then allow institutions to deploy targeted supports aligned with individual profiles—for example, pre-emptive adaptation skill workshops for high-GPA/low-resilience entrants or science foundation bolsters for applicants with high interpersonal scores but limited formal background in biology/chemistry [35]. Ultimately, optimizing for both performance sustainability and well-being requires recognizing that neither admissions criteria nor intra-program support alone are sufficient levers; it is their interaction over time, with evolving stress landscapes, which shapes the emergent equilibrium between thriving, surviving, or attiring within veterinary education pathways.

## 7. Institutional Interventions and Programmatic Responses

In response to the growing recognition of mental health challenges among veterinary students, educational programs have begun adopting structured interventions that promote psychological well-being and resilience. Evidence indicates that embedding wellness services within veterinary schools enhances access to support, with on-site mental health professionals fostering greater counseling engagement and proactive coping behaviors [30]. Parallel curricular innovations have yielded encouraging results; for instance, leadership and wellness modules have been shown to reduce perceived stress and even correlate with improved academic outcomes [16]. Integrating emotional support mechanisms within clinical training also appears beneficial. Compassion-focused initiatives, such as “Compassion Rounds,” help students process ethically challenging experiences and mitigate burnout [42]. Systematic evidence further suggests that even brief interventions (such as mindfulness, resilience, or study-skills workshops) can significantly decrease stress and anxiety levels following participation [25]. While many studies are limited by short follow-up durations, the overall literature highlights that well-being initiatives can be embedded feasibly within existing curricula without compromising academic standards. Recent reviews also emphasize that enduring impact depends on sustained, institution-wide strategies integrating resilience-building, mentorship, and normalization of help-seeking behavior [43]. Collectively, these findings underscore that comprehensive and continuous well-being framework, rather than isolated interventions, are essential for preparing veterinary graduates who are both competent and psychologically resilient [44].

## 8. Concluding Remarks

Several limitations within the current evidence base warrant acknowledgment. First, most empirical studies informing this synthesis employ cross-sectional methodologies, restricting conclusions about causality and long-term outcome prediction. Second, substantial heterogeneity exists in how non-cognitive variables are defined and measured, reducing cross-study comparability and limiting the development of standardized admissions models. Third, the majority of available data derive from North American, European, and Australasian veterinary programs. Educational structures, financial burdens, and cultural attitudes toward help-seeking differ internationally, and therefore generalizability to other educational systems requires cautious interpretation. Expanded research in underrepresented regions would strengthen the global applicability of these findings.

This mini-review highlights that veterinary education is a high-demand environment in which academic rigor, clinical responsibilities, and psychosocial pressures converge. These combined demands directly affect student performance, mental health, and long-term professional sustainability. Stress within veterinary training is not incidental; it is shaped by curricular structure, assessment intensity, financial burden, and institutional culture.

Traditional cognitive indicators such as GPA and standardized test scores remain useful predictors of early academic performance. However, they do not capture essential attributes such as resilience, adaptability, empathy, motivation, and communication skills. These qualities are critical for persistence and clinical competence. Expanding admissions frameworks to incorporate validated non-cognitive assessments could therefore improve selection processes. Development of standardized holistic models should be considered by academic leaders and policy makers.

Curricular design also plays a central role in shaping student stress. Overloaded schedules, abrupt transitions into clinical training, and limited mentorship increase strain and may compromise learning. Institutions can mitigate these risks by adjusting curricular pacing, maintaining appropriate faculty-to-student ratios, and embedding resilience training and wellness initiatives within the formal curriculum.

Financial vulnerability and social isolation further compound academic pressures. Scholarships, accessible counseling services, structured mentorship, and peer-support programs provide important protective mechanisms. Coordinated strategies across admissions policy, curriculum design, and institutional support systems are necessary to reduce burnout, improve retention, and prepare graduates for the complex demands of contemporary veterinary practice.

While this review does not present original empirical data, its contribution lies in integrating admissions research, stress psychology, and institutional intervention literature into a unified conceptual framework. By synthesizing these domains, the manuscript aims to clarify interaction patterns and identify actionable leverage points for veterinary education systems. Future empirical studies should test these proposed relationships prospectively across diverse educational settings.

## Figures and Tables

**Table 1:** Stressors and Coping Approaches Among Veterinary Students: Challenges, Impacts, and Adaptive Strategies.

Domain	Examples/Mechanisms	Observed/Potential Impacts	Limitations/Considerations	References
Academic Stressors	High content volume; frequent high-stakes exams; misaligned lecture-lab sequences; intensive clinical rotations	Cognitive overload; diminished retention; performance anxiety; increased risk of burnout	Curricular pacing reforms and supportive teaching approaches may mitigate strain	[4,8,13,14].
Non-academic Stressors	Financial debt; relocation isolation; competitive cohort culture; interpersonal skill deficits	Depressive symptoms; social withdrawal; reduced resilience; impaired professional identity formation	Access to scholarships, mentorship, and counseling critical for mitigation	[5,6,15].
Intersecting Stressors	Ethical dilemmas (euthanasia); client communication challenges; faculty shortages limiting mentorship	Moral distress; reduced self-efficacy; slower clinical skill acquisition	Ethical training and structured mentorship can buffer impacts	[12,16,17,18]
Coping–Physical Activity	Regular exercise and sports	Lower depressive symptoms; improved concentration	Adherence declines during peak academic load	[19,20]
Coping–Study Organization	Structured study planning	Reduced cramming; better retention	Often requires initial mentoring to adopt	[21]
Coping–Peer Support	Participation in peer-support groups	Greater belonging; exchange of strategies	Group dynamics and confidentiality may pose risks	[22,23].
Coping–Stress Relief	Humor use	Immediate mood lift; tension diffusion	Limited for chronic stress; risk of masking problems	[15]
Coping–Mindfulness	Guided mindfulness practice	Reduced anxiety before assessments	Cultural attitudes influence uptake	[24,25]
Coping–Hobbies	Engagement in hobbies outside school	Preserves identity facets; reduces burnout	Easily eroded during workload surges	[6,26]
Coping–Faculty Support	Seeking advice from faculty advisors	Clarifies expectations; tailored guidance	Dependent on advisor availability and rapport	[27]

**Table 2:** Interaction trends between admissions predictor types, dominant stressors experienced, and observed outcome trajectories in veterinary students.

Admissions Predictor Type	Common Amplifying/Triggering Stressors	Observed Early Outcomes	Longer-Term Trends (If Unmitigated)	References
High GPA/GRE profile	Ambiguity in clinical settings; ethical decision pressures	Strong didactic performance; high initial confidence	Possible erosion of advantage if adaptability skills lag; heightened anxiety during role transition	[14,41]
Strong non-cognitive (resilience/adaptability)	Dense theoretical load; high-frequency assessments	Smoother adjustment to new formats; stable peer integration	Gradual improvement academically if technical gaps are addressed; sustained lower burnout indicators	[10]
Mixed moderate scores across domains	Financial burden; relocation isolation	Variable academic start; dependent on access to peer/faculty support	Polarized outcomes, improvement with targeted intervention or decline under compounded stress	[3]
Low cognitive + low measured resilience	All stressor categories proportionally impactful	Elevated remediation risk; social withdrawal possible early on	High attrition probability without intensive multifaceted support	[8,33]

## Data Availability

No new data were created or analyzed in this study.
